# The Influence of Proportion Dominance and Global Need Perception on Donations

**DOI:** 10.3389/fpsyg.2022.800867

**Published:** 2022-06-02

**Authors:** Danit Ein-Gar, Amir Give’on

**Affiliations:** ^1^The Marketing Department, Coller School of Management, Tel-Aviv University, Tel Aviv, Israel; ^2^Fund More Good Foundation, Los Angeles, CA, United States

**Keywords:** charity, donations, prosocial, help-giving, proportion dominance, choice, reference point, need perception

## Abstract

Many donation-raising platforms request that first-time donors choose the charitable causes they most care about so that future campaign recommendations can best match donors’ charitable preferences. While matching charitable campaigns to donors’ reported preferences has its benefits, little is known about other effects that choosing charitable causes may evoke. We focus on how choosing charitable causes influences charitable behavior. We find two effects of the number of charitable causes donors choose on their subsequent charitable behavior. In studies 1 and 2, we show that a reference number of the maximum charitable causes donors can choose has a negative effect on charitable behavior. A small (versus large) reference number yields a greater likelihood to donate and a higher donation amount. This effect is aligned with the proportion dominance rationalization. In studies 3 and 4, we show that the number of charitable causes donors voluntarily choose as important to them is positively associated with subsequent charitable behavior. This association is mediated by global need perception. As the number of causes donors choose increases, donors experience an escalation in their perception of global neediness, which in turn motivates their willingness to donate and the donation amount. In Study 5, we show how the two effects together shape charitable behavior. These effects are observed while controlling the donors’ inherent prosocial attitudes toward help giving. With more than 1.5 million registered non-profit organizations operating in the United States (National Center for Charitable Statistics, 2019), it has become almost impossible for donors to easily choose which charitable campaigns to support. Online charitable fundraising platforms (e.g., One Today by Google, Round Up, and Charity Miles), websites (e.g., AmazonSmile) and crowdfunding platforms (e.g., Fundly, JustGiving, and GoFundMe) try to ease donors’ search and decision processes by offering them personalized charitable options. First-time donors are asked to indicate the charitable causes they care most about, and then asked to donate to charitable campaigns that best match their preferences. Interestingly, little is known about how this initial stage of choosing charitable causes influences subsequent donation behavior. In this research, we ask how choosing the charitable causes one cares most about influences subsequent response to a charitable appeal. Obviously, the mere selection of preferred causes enables charities to offer personalized campaigns and create a better fit between non-profits and donors, which has a generally positive effect on charitable giving. However, in this research we focus on an overlooked aspect of these practices. We examine how the number of charitable causes donors indicate as important to them influences their donation giving. We test two opposite effects: the *proportion dominance effect*, an effect driven by prior research, and *the global need perception effect*, a new effect identified in this article. Both effects are driven by the number of causes donors choose.

## Introduction

Donors constantly make choices; they choose charitable causes (e.g., world hunger, human rights), charitable organizations (e.g., savethechildren.org, childfund.org), charity-raising platforms (e.g., gofundme.com, fundly.com), as well as specific donation recipients (e.g., a needy child in Africa, a needy immigrant in the United States).

Research on the choice of donation recipients shows that when donors need to choose between helping a group or helping a single person, they prefer to donate to the group (Kogut and Ritov, 2005; Ein-Gar et al., 2018), suggesting that helping many is valued more than helping one. However, this size-valuing effect may be sensitive to group size. For minimal groups, such as a group of three needy individuals, the effect was not replicated (Erlandsson, 2021). When the choice is between two single people in need, donors may face a moral dilemma between the wish to help and the wish to do so in a fair manner. When fairness concerns arise, 35–50% of prospective donors decide to avoid choosing altogether (Ein-Gar et al., 2021). However, if the two people in need differ on some attribute such as gender or physical attractiveness, then donors are more likely to choose one over the other, basing their decision on peripheral attributes such as beauty (Cryder et al., 2017) or gender (Bareket et al., 2022; Ein-Gar et al., 2022).

When donors need to choose one of several charitable organizations or campaigns, familiarity becomes a key factor. Familiar charities and campaigns are chosen more often than non-familiar ones (Soyer and Hogarth, 2011). Offering donors choice sets that vary across many options (8 vs. 16; 7 vs. 13) does not increase donation likelihood (Soyer and Hogarth, 2011). It may even reduce the likelihood of helping because of decision difficulty (Carroll et al., 2011).

The above studies demonstrate that when donors need to choose one donation recipient (a specific individual, a campaign, or an organization) certain underlying processes come into play. However, different underlying processes take place for different types of choices. For example, donors may feel that it is unfair to choose one needy individual over another similarly needy individual, but may not feel it is unfair to choose one charitable cause (e.g., immigrants) over another similar charitable cause (e.g., minorities). Furthermore, these processes are unique to choosing a specific recipient that will directly benefit from the decision. However, donors sometimes make sequential decisions, where the first decision involves a choice that does not directly influence a recipient. For example, donors may begin by choosing a general charitable cause (e.g., helping children from underprivileged backgrounds), then choose an organization (e.g., worldvision.org), and only then decide to help a specific recipient (e.g., donating to a specific child who can’t afford school supplies). In these cases, other processes may drive charitable behavior. Furthermore, in most of the aforementioned studies, the underlying process was relevant to cases where donors were obligated to choose a single option. It is, therefore, essential to broaden the research investigation of how choices influence donation behavior to choices that do not directly influence a specific recipient and to decisions that involve choosing more than one option (as in the case of choosing charitable causes). These broader choice settings may reveal new underlying processes that influence charitable behavior.

The current research focuses on how an initial stage of choosing several charitable causes (rather than choosing a single donation recipient) influences subsequent donation-giving behavior. Specifically, we ask participants to choose several charitable causes they care most about and test whether the number of charitable causes they choose influences their donation giving. We explore two effects driven by the number of charitable causes chosen. We first test how giving participants a number referencing the maximum number of causes they need to choose influences their subsequent donation giving (studies 1 and 2). We find that a large reference number (being asked to choose 8 or 10 causes) yields fewer donations than a small reference number (being asked to choose 4 or 5 causes). These findings are in line with prior research on the effect of proportion dominance. We then test whether the actual number of causes that donors can voluntarily choose from (being asked to choose up to 7 causes, without manipulating a fixed reference number) is related to their donation behavior (studies 3 and 4). We find that the more causes donors select as important to them, the more they are likely to donate. Our reason is that the voluntary process of choosing more causes activates an escalation of global need perception, which in turn increases donation giving. Finally, we show how both effects together influence donation giving (Study 5).

### Reference Number and the Proportion Dominance Effect

Proportion responding occurs when individuals make decisions based on a proportion inference rather than absolute quantity (Mata, 2016). Thus, for example, individuals consume more quantities of food when they are offered food from a large bowl versus a small bowl. The quantity consumed is assessed as a portion of the entire bowl. As a result, the same quantity is perceived as proportionally smaller when the bowl is large compared to when it is small. A similar phenomenon, termed the proportion dominance effect, has been found in risk assessment and help giving (Baron, 1997; Slovic et al., 2002). Individuals are more likely to help victims that are part of a small group than to help the same number of victims that are part of a large group (Bartels, 2006; Erlandsson et al., 2014, 2015). This effect is attributed to an assessment of the utility of the help given. Individuals perceive higher utility when they help 10 out of 11 people in need than when they help 10 out of 1,000 people in need, despite the fact that in both cases the absolute number of people being helped is the same (Erlandsson et al., 2014). The effect was even observed when the proportion was higher yet the absolute quantity was lower, such that individuals prefer saving 10 out of 10 lives over saving 11 out of 100 lives (Mata, 2016). The smaller the reference group, the greater the perceived impact of the help giving (Friedrich and Dood, 2009). Some studies suggest that this effect is influenced by mental representations. When individuals think about their decision in terms of a group rather than in terms of many individuals, the effect strengthens because helping a large proportion of a whole unit (i.e., a group) is more satisfying than helping a small proportion of many units (Bartels and Burnett, 2011). This effect was found for decisions involving helping not only humans but also non-humans such as animals (Bartels and Burnett, 2011).

Following this logic, we propose that when individuals first choose the charitable causes they care about and then consider helping a campaign related to one of the causes, they evaluate the impact of their donation in reference to the number of causes they care about. The smaller the reference number of causes, the more impactful the donation feels. Therefore, helping one cause (by supporting its campaign) out of three important causes would be valued more than helping one cause out of seven important causes.

According to this reasoning, we hypothesize that the smaller the reference number of charitable causes donors consider as important to them, the greater their willingness to donate and their donation amount.

### Number of Causes Selected and the Global Need Perception Effect

Informing prospective donors about causes, non-profits, and groups or individuals in need of help is a prerequisite for donation giving. According to the literature review by Bekkers and Wiepking (2011), need awareness is the first mechanism that drives donation giving. Once aware of a need, donors assess its extremeness before deciding whether to reach out and help. Need assessment can be based on such aspects as the helplessness of the victim, as in the case of children (Lee and Feeley, 2016); the severity or urgency of the cause, as with organ donations (Tsai et al., 2000); or the magnitude of the need, as in the case of humanitarian crises (Bennett and Kottasz, 2000; Huber et al., 2011). The more individuals perceive the intensity of the need, the more likely they are to provide help (Wagner and Wheeler, 1969; Schwartz, 1974).

Most, if not all, research on need perception has focused on a specific need related to a specific cause, event, group, or individual. Thus, for example, the well-established effect of the identifiable victim (Kogut and Ritov, 2005) suggests that a specific individual with a specific, vivid need raises more charitable responsiveness than statistical victims or charitable organizations (Kogut and Ritov, 2011; Ein-Gar and Levontin, 2013; Lee and Feeley, 2016). This effect, which is driven by the salience of a single person in need, diminishes when donors become aware of others who have a similar need yet are not given help (Västfjäll et al., 2015; Ein-Gar et al., 2021).

In this research, we propose that in addition to awareness of a specific need, there also may be an awareness of the overall neediness in the world. We define “global neediness perception” (hereafter GNP) as a reflection of donors’ perception regarding the extent to which there are few or many causes in the world that need charitable support. For example, some individuals may feel that geopolitical and environmental changes (e.g., polarized societies, global warming, industrialized pollution, and global pandemics) have increased the number of social and environmental causes that need charitable support. Others might feel that social and technological advances such as social and environmental movements and advancements in agrotechnology, biotechnology, and medicine offer solutions to many social and environmental problems and that overall need in the world is declining.

Global neediness perception may reflect a relatively stable individual difference. Thus, for example, in an online survey among US participants (Prolific, *n* = 501, M_*age*_ = 40, November 2021), we examined the relationship of GNP with other individual differences. Specifically, participants read: “Some people feel that there are many important social issues in need of charitable support, while others feel that everything narrows down to a few general important issues in need of support.” Participants then reported their estimation of the number of social issues in need of support in the world on a 5-point scale ranging from 1- very few causes to 5- numerous causes. Participants also answered the Helping Attitudes Scale (HAS; 20 items, α = 0.89; Nickell, 1998) and the Fear of COVID-19 scale (FCV-19; 7 items, α = 0.91; Ahorsu et al., 2020). The order of all measures was randomized. GNP was positively correlated with differences in individuals’ concerns regarding the COVID-19 pandemic (*r* = 0.195, *p* < 0.001) and with a prosocial personality reflected by general attitudes toward help giving (*r* = 0.33, *p* < 0.001).

However, as with many other individual differences, this perception can be temporally altered. For example, in two unrelated studies asking US participants to donate in different contexts, participants also reported their GNP at the end of the studies (same introduction to GNP as in the previously described study; single item, scale 1–7). One study (Prolific, *N* = 440, *M*_*age*_ = 33) was conducted during October 2019, prior to the COVID-19 outbreak, which started in November 2019. The other study (Prolific, *N* = 395, *M*_*age*_ = 31), was conducted during September 2021 while the global pandemic was ongoing and induced participants to think about the pandemic and its implications. We found that participants who reflected on the pandemic and its implications reported significantly higher GNP (*M* = 6.03, *SD* = 1.04) than participants whose mindsets were not focused on the pandemic (*M* = 3.79, *SD* = 1.07); these scores are significantly different [*t*(833) = 30.62, *p* < 0.001].

In the current research, we test whether choosing several charitable causes is related to the prospective donors’ GNP. We suggest that as donors consider the different charitable causes they can support and decide which ones they care about most, they also consider the needs of each cause. Regardless of which causes they choose, as the number of causes chosen as important and in need of support rises, donors reflect on different needs worldwide and experience an escalation in their GNP. We test whether the higher the GNP results in a greater willingness to reach out and help as expressed by the willingness to support a charitable campaign and donate greater amounts.

### Overview of the Present Research

We conducted five studies. The first two studies test how a fixed reference number of small vs. large important charitable causes influences donation giving and find a negative effect, such that a small number of charitable causes increases donation likelihood more than a large number of causes. The second two studies test how the actual rise in the number of causes selected (without manipulating a fixed reference number) relates to GNP and donation giving and find a positive relationship, such that the more charitable causes donors choose, the higher their GNP and the more likely they are to donate. In the last study, participants are given a reference number of charitable causes yet can still make a varied choice of causes, and we test both effects together.

We report in our studies how we determined our sample size, all manipulations, and all measures. No data were excluded from analyses.

## Study 1

The goal of Study 1 was to test how asking participants to select either a small or large number of charitable causes important to them influences their GNP and their subsequent donation giving. In this study, participants were asked to choose either a fixed small number (4) or a fixed larger number (8) of charitable causes from a list of causes and afterward indicate their willingness to donate to a campaign related to one of their chosen causes. According to the proportion dominance effect, we hypothesize that participants referenced with a small number of causes (4) will be more likely to donate than participants referenced with a large number of causes (8). However, we did not have a prediction on whether obligating participants to choose a small or large number of causes will influence GNP.

Considering the different charitable causes is likely to elicit general thoughts about help giving. Such thoughts may activate heightened perceptions about the importance of helping and the positive implications of help giving, which in turn may influence more charitable behavior. To account for such an effect, Study 1 measured participants’ attitudes toward help giving. Some participants reported their attitudes toward helping before the reference number manipulation and the decision to donate, while others reported their attitudes after the decision to donate. This was done to test whether this measure reflects a stable individual difference in attitudes toward helping. We incorporate this measure into the model as a covariate to test the effect of the reference number of charitable causes on donation giving, above and beyond dispositional attitudes toward helping.

### Materials and Methods

Two hundred and seven students (*M*_*age*_ = 24.67, 60.3% female) recruited from a university online pool participated in this study in exchange for course credit and entered a raffle with 20 prizes of 50ILS each (equivalent to $15).

In this and all subsequent studies, we strived to achieve sample sizes over *n* = 90, which are sufficient to detect medium-sized effects of *r* = 0.30 with a power of 0.90 and 0.05 Type I error probability.

Participants were introduced to a description of a charity app named “CausePick.” They read that the app sends its users monthly personalized recommendations of charitable campaigns. Each month users choose a campaign to which “CausePick” automatically transfers their donation. Participants were asked to assume they are entering the app for the first time and needed to indicate their charitable preferences, according to which the app will generate personalized recommendations for charitable campaigns. All participants received the same list of 17 causes (hunger, education, minorities, environment, etc.) and were asked to choose the causes they most care about. Participants were randomly assigned to two experimental conditions. In the small reference number condition, participants were instructed to choose 4 causes (small condition), while in the large reference number condition participants were instructed to choose 8 causes (large condition). On the following page, participants indicated their intention to support a charitable campaign, assuming it fits their charitable preferences. Participants responded on a 7-point scale (1 = no chance I will donate, 7 = I will definitely donate). Next, participants indicated the amount they would be willing to donate to the campaign if they won the prize raffle, with answers recorded on a 50-point scale (0 = I will not donate any amount, 50 = I will donate all). On a subsequent page, participants indicated their GNP. This measure is similar to the one reported in the introduction. Specifically, they read: “*Some people feel that there are many important social issues in the world that need the support of donations, while others feel that everything converges into a small number of important social issues that need charitable support. What, in your opinion, is the scope of all the social issues that need the support of donations in the world?*” Answers were given on an 11-point Likert scale (1 = very few, 11 = plentiful).

In this study participants also completed the Helping Attitude Scale (HAS; 20 item, α = 0.77; Nickell, 1998) measuring beliefs, feelings, and behaviors related to helping (same measure as mentioned in the “Introduction” section). At random, half of the participants completed the scale at the beginning of the study before reading about the charity app and making their donation decision; the other half completed the scale after reading about the app, choosing their causes, and making the donation decision. Finally, participants indicated demographics such as age, gender, and mother tongue. (For more details see Supplementary Appendix A). The data from studies 1, 2, 4, and 5 and the studies reported in the introduction section can be accessed at: https://osf.io/8gr6n/?view_only=b0703de9c9de4710ac3b48a85bbd54ec.

### Results

As a preliminary step, we tested the effect of HAS presentation order and found it to be non-significant [*t*(205) = 0.08, *p* = 0.94]. We tested the interaction effect of HAS presentation order and the reference number of charitable causes condition on the two independent variables. We found no significant effects (see results in Supplementary Material). This suggests that thoughts about charitable causes or the number of charitable causes people think about do not influence the helping attitude or interact with helping attitudes, presumably because helping attitudes reflect a stable personality attribute.

Descriptive statistics and intercorrelations of the study variables appear in Table 1. As seen in the table, HAS was significantly correlated with both GNP and the dependent measures and, therefore, was controlled for in the subsequent analysis.

**TABLE 1:** Mean, std. deviation, and intercorrelations of Study 1 variables (*N* = 207).

Variable	Mean	SD	1	2	3	4	5
Reference number of charitable causes (0 = Four; 1 = Eight)	1.48	0.50	−				
WTD (Single item; 1–7)	4.52	1.88	−0.16*	−			
Donation amount (0–$50)	24.18	16.59	−0.15*	0.63**	−		
GNP (Single item; 1–11)	7.33	2.51	0.03	0.30**	0.32**	−	
HAS	3.33	0.25	02	0.24**	0.19**	0.22**	−

*p < 0.05; **p < 0.001.

The manipulated reference number of charitable causes did not have an effect on GNP, but did affect the dependent variables: Participants presented with a smaller reference number of causes (4) were more willing to donate (*M* = 4.81, *SD* = 1.73) and indicated donations of greater amounts (*M* = 26.56, *SD* = 16.31) than participants with a larger reference number of causes (8; willingness to donate: *M* = 4.20, *SD* = 2.0; donation amount: *M* = 21.52, *SD* = 16.57). These differences are significant [willingness to donate: *t*(205) = 2.34; *p* = 0.02; donation amount: *t*(205) = 2.19; *p* = 0.03]. GNP was significantly correlated with both of the dependent variables: *r* = 0.30 with willingness to donate and *r* = 0.32 with donation amount (both *r*’s *p* < 0.001).

The hypothesized relations between research variables were tested as a path model using Mplus Version 8.6 (in this and all subsequent studies; Muthén and Muthén, 2017). This saturated model fitted the data perfectly, with χ^2^(0) = 0.00.

**FIGURE 1:**
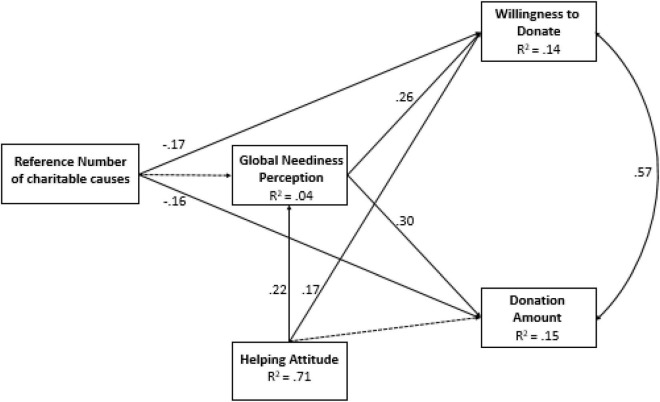
Path model from reference number of charitable causes to willingness to donate and donation amount, mediated by GNP and controlled for HAS (Study 1). The values along the paths are standardized regression coefficients (betas), and correlation is shown along the double-arrow curve. The broken lines indicate statistically non-significant paths (*p* ≥ 0.05).

The results (Figure 1) show that the reference number of charitable causes had negative effects on both dependent variables: willingness to donate (*p* = 0.007) and donation sum (*p* = 0.013); however, these effects were not mediated by GNP (both *p*’s = 0.775). Controlling for HAS, GNP was significantly and positively associated with both willingness to donate and the donation amount (both *p*’s < 0.001).

### Discussion

The results of Study 1 show that when donors’ reference number of charitable causes is small, they are more likely to donate and to donate greater amounts than when their reference number of charitable causes is large. These findings are in line with proportion dominance reasoning, according to which donors who help one cause out of four feel that their help is more meaningful and as a result have a stronger motivation to donate than donors who help one cause out of eight.

The small versus large reference numbers of charitable causes did not change participants’ perception of neediness in the world or their attitudes toward helping. One of the reasons could be that participants were obligated to choose a fixed number of causes (either 4 or 8) rather than having the liberty to choose their own number of causes. In the next study, we aim to test the robustness of these findings by replicating the results with a different sample and slightly different reference numbers.

## Study 2

The main goal of Study 2 was to replicate the results of Study 1 with a non-student sample and with different reference numbers of charitable causes. To that end, the design of this study was similar to that of Study 1, with participants instructed to select a fixed small or large number of charitable causes. We tested how these reference numbers influence subsequent donation giving. According to the proportion dominance effect, we hypothesize that participants referenced with a small number of causes will be more likely to donate than participants referenced with a large number of causes. Following the results of Study 1, we do not expect that the number of charitable causes that participants are obligated to select will influence their GNP or HAS.

An additional goal of this study was to provide further validity for the GNP measure. Therefore, in this study, GNP was measured with four items (rather than 1), and we test its relation to two other individual differences constructs. Specifically, we test whether a general high or low optimistic nature (Scheier et al., 1994) changes how one experiences GNP and whether concerns with the COVID-19 pandemic and its implications (Ahorsu et al., 2020) are positively related to GNP. These results will replicate initial findings that were reported in the Introduction (see page 9), but with a 4-item measure of GNP.

### Materials and Methods

Five hundred and one adults (*M*_*age*_ = 38.8, 49.5% female) recruited through Prolific participated in this study in exchange for $0.8 payment and entered a raffle with a $20 prize.

The procedure was the same as in Study 1 with a few changes: First, the reference numbers were 5 (small condition) and 10 (large condition). Second, we added two individual- differences measures at the end of the study: dispositional optimism assessed by the Life Orientation Test (LOT; 10 items, α = 0.73; Scheier et al., 1994) and the Fear of COVID-19 scale (FCV-19; 7 items, α = 0.90; Ahorsu et al., 2020). Based on initial results (see “Introduction” section) we expected FCV-19 to positively correlate with GNP. However, we did not expect GNP to correlate with LOT. Third, we measured GNP with more items. In addition to the original item in Study 1 (measured on a 5-point scale), we added three items: *Please state how you perceive the global neediness in the world today* (1 = Almost no global neediness, 7 = Excessive global neediness); *In your opinion, what is the scope of social and environmental issues worldwide which require charitable support* (1 = Few issues, 7 = Many issues); *What is your opinion about the social and environmental issues worldwide which require charitable support* (1 = Insignificant issue/s, 7 = Significant issue/s). All four items were averaged into a single GNP score (α = 0.85). As in Study 1, we counterbalanced HAS, such that half of the participants completed it at the beginning of the study and half at the end (α = 0.88). Finally, participants indicated demographics such as age, gender, and mother tongue (For more details see Supplementary Appendix B).

### Results

As in Study 1, we first tested for an order effect for HAS. Unlike in Study 1, we found that participants who completed HAS at the end of the study reported higher scores (*M* = 3.98, *SD* = 0.50) compared to participants who completed the scale at the beginning of the study [*M* = 3.83, *SD* = 0.53, *t*(499) = −3.34; *p* < 0.001], suggesting that in the present study, the mere thought of charitable giving increased attitudes toward helping. To test if the reference number of charitable causes influenced HAS, we conducted a *t*-test only among participants who completed HAS at the end of the study (*N* = 254). We did not find significant differences between participants in the small condition (*M* = 3.97, *SD* = 0.46) and participants in the large condition [*M* = 3.99, *SD* = 0.53, *t*(252) = −0.25, *p* = 0.80]. Thus, attitudes toward helping were not influenced by the reference number manipulation. Finally, we tested the interaction effect of HAS presentation order and the reference number of charitable causes condition on the two independent variables. We found no significant effects (see results in Supplementary Material).

For descriptive statistics and intercorrelations between the study variables, see Table 2. As Table 2 shows, GNP is positively correlated with HAS (*r* = 0.43, *p* < 0.001); GNP is also positively correlated with FCV-19, although to a lesser extent than with HAS (*r* = 0.11, *p* = 0.02), and does not correlate with LOT (*r* = 0.07, *p* = 0.11). These results suggest that individuals who feel there are many issues in the world that need charitable support also hold positive attitudes toward helping and are concerned with the negative implications of COVID-19, However, they are not highly pessimistic or optimistic in nature.

**TABLE 2:** Mean, std. deviation, and intercorrelations of study 2 variables (*N* = 501).

Variable	Mean	SD	1	2	3	4	5	6	7
Reference number of charitable causes (0 = Five; 1 = 10)	0.49	0.50	−						
WTD (Single item;1–7)	4.36	1.59	−0.10*	−					
Donation amount (0–$20)	8.68	6.21	−0.11*	0.44**	−				
GNP (4 items; 1–7)	5.19	0.99	0.01	0.25**	0.24**	−			
HAS	3.91	0.52	0.02	0.42**	0.39**	0.43**	−		
Optimism	2.59	0.62	–0.02	0.14**	0.13**	0.07	0.27**	−	
Fear of COVID-19	1.08	0.83	–0.01	0.14**	0.07	0.11*	0.12**	−0.14**	−

*p < 0.05; **p < 0.001.

The reference number of charitable causes was not a significant predictor of GNP (*p* = 0.83), but it was significantly related to the dependent measures: Participants with a smaller reference number of charitable causes (5) were more willing to donate (*M* = 4.51, *SD* = 1.59) and indicated donations of greater amounts (*M* = 9.35, *SD* = 6.14) than participants with a larger reference number of charitable causes (10; willingness to donate: *M* = 4.20, *SD* = 1.58; donation amount: *M* = 8.0, *SD* = 6.21). These differences are significant [willingness to donate: *t*(499) = 2.15; *p* = 0.032; donation amount: *t*(499) = 2.45; *p* = 0.015].

**FIGURE 2:**
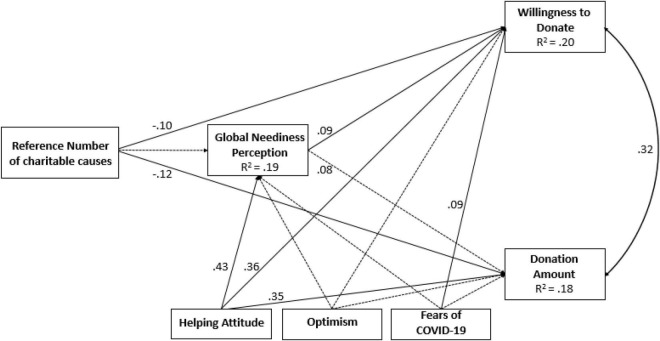
Path model from reference number of charitable causes to willingness to donate and donation amount, mediated by GNP and controlled for HAS, optimism and fear of COVID-19 (Study 2). The values along the paths are standardized regression coefficients (betas), and correlation is shown along the double-arrow curve. The broken lines indicate statistically non-significant paths (*p* ≥ 0.05).

The path model of the theoretical relations between research variables fit the data well, with χ^2^(3) = 0.56, *p* = 0.90; its results appear in Figure 2.

The reference number manipulation has a direct negative effect on willingness to donate (*p* = 0.011) and the donation amount (*p* = 0.004); however, these effects are not mediated by GNP (both *p*’s = 0.965). Controlling for HAS, LOT, and FCV-19, GNP is related to willingness to donate (*p* = 0.046) and is related in the expected direction to donation amount yet does not reach significance (*p* = 0.060).

We conducted another path model without the covariates of optimism and fear of COVID-19 (similar to the model in Study 1); the results are almost identical (for the full model description see Supplementary Material).

### Discussion

The results of Study 2 echo the results of Study 1 in showing that the reference of a small number of charitable causes induces a higher likelihood to donate as well as higher donation amounts compared with the reference of a larger number of charitable causes. According to the proportion dominance rationalization, this effect can be explained by donors’ sense of contribution impact. Donating to one cause out of five could be experienced as more impactful than donating to one cause out of ten. Therefore, when referenced with a smaller rather than larger number of charitable causes, donors perceive their help as more meaningful and, as a result, the motivation to donate increases. Furthermore, as in Study 1, results show that the smaller vs. larger reference number did not influence other antecedents of donation giving such as GNP, attitudes toward helping, or concerns regarding the COVID-19 pandemic.

Taken together, the results of studies 1 and 2 suggest that asking donors to choose a fixed small number rather than a larger number of charitable causes they care most about increases their overall prosocial behavior, as expressed by greater willingness to donate and higher donation amounts. However, in both studies, donors were compelled to choose an exact number of charitable causes (either small or large). In the next studies, we ask donors to choose the charitable causes they most care about, but without forcing them to choose an exact number. This “freedom of choice” allows other underlying motivations to kick in. We aim to test whether the number of causes donors voluntarily choose influences their charitable behavior.

Although we allow variation in the number of causes donors choose, we provide a maximum number in order to minimize task depletion or choice overload effects.

## Study 3

The goal of Study 3 was to test whether and how the number of causes chosen influences charitable behavior in a natural setting. We used data from an online fundraising platform that offers its users monthly personalized recommendations for charitable campaigns. The web-based platform designated for raising donations was established in November 2016 and was active until the end of 2018.^1^

Users registering on the platform for the first time chose the charitable causes they care about most and were presented with three personalized charitable campaigns matching their charitable preferences. Users picked one campaign to which they wished to donate and then were transferred to a payment page where they indicated the amount they wished to donate.

In this study, we focused on users’ charitable behavior the first time they entered the platform and in reference to the first donation decision they made. We tested whether the number of charitable causes users chose predicted their willingness to support a charitable campaign and affected their donation amount.

### Materials and Methods

When users entered the platform for the first time (i.e., the home page), they read a description about the platform and how it works. On the next page, users viewed 24 charitable causes (presented by title and icon) and chose up to 7 causes they care about the most. The number of causes they chose served as our independent measure. After making their choice, users were transferred to a new page on which they were offered three personalized charitable campaigns. Users could decide to donate to one of the three campaigns or exit the platform. Whether or not users clicked on one of the campaigns, thereby indicating an initial willingness to donate, served as our first dependent measure. Users who clicked on the campaign were transferred to the payment page on which they indicated the amount of donation they wished to make for their chosen charity. The amount users donated served as our second dependent measure. On the final page, users provided their personal payment details, and their donation was transferred to their preferred charitable campaign (For more details see Supplementary Appendix C).

All our analyses are based on data provided by 480 users who entered the platform and indicated the charitable causes important to them. The data for each user included the number of causes chosen (up to 7), willingness to donate as indicated by whether the user clicked on a campaign they wish to support, and the donation amount users indicated on the payment page (ranging from 0 to $108).

### Results

Descriptive statistics and intercorrelations between the study variables are shown in Table 3.

**TABLE 3:** Mean, std. deviation, and intercorrelations of study 3 variables (*N* = 480).

Variable	Mean	SD	1	2	3
Number of charitable causes chosen (up to 7)	3.80	2.18	−		
WTD (1 = Choose a campaign; 0 = Did not choose a campaign)	0.36	0.48	0.14**	−	
Donation amount (0–$108)	2.21	12.61	0.08	0.23**	−

**p < 0.001.

#### Willingness to Donate

Users who clicked on a charitable campaign, thus indicating their willingness to donate, were coded as 1, while users who did not select a campaign were coded as 0. We conducted a logistic regression to test if the number of causes chosen served as the predictor of whether donors clicked on one of the campaigns or not. Results show that the number of causes chosen serves as a positive and significant predictor of willingness to donate—that is, initial intention to support a specific charitable campaign (*B* = 0.135, *S.E*. = 0.044; Wald = 9.38, *p* = 0.002).

#### Donation Amounts

We conducted a regression on the entire sample to test whether the number of causes chosen predicted the amount of money donors decided to donate. Results are in the expected positive direction; however, they did not reach significance (*B* = 0.46, *S.E*. = 0.26, β = 0.08, *t* = 1.75, *p* = 0.08).

### Discussion

Results of Study 3 show that the more charitable causes users voluntarily chose as important to them (without manipulating a reference number of causes), the greater the likelihood to support a campaign. However, in this study, an increase in the number of causes chosen did not yield a significant increase in the amount of money users donated.

This study, although based on actual behavior in a natural field setting, is not without limitations. First, users entering the app were not randomly selected; most users *a priori* indicated some interest in giving to charity. Hence, there may be a selection bias. Second, the results, although in the same direction for both donation-behavior measures, were found to be significant only for willingness to donate but not for the donation amount. One possible explanation could be attributed to restricted variance. The app recommended certain donation amounts, which could have reduced the variance and suppressed a significant relationship from emerging. Third, the data provided evidence for the main effect without providing any insight as to why these relations emerge and whether they are related to an escalation in GNP. Finally, it is possible that the number of charitable causes chosen is merely an expression of individuals’ attitudes toward helping. The more users demonstrate positive helping attitudes, the more charitable causes they choose and the more willing they are to donate. Therefore, it is important to control for individual differences in helping attitudes when exploring the link between number of charitable causes chosen and donation behavior, as was done in studies 1 and 2. The next studies aim to expand our understanding of the relationship between the number of charitable causes chosen and charitable behavior.

## Study 4

The goal of Study 4 was to test the relationship between the number of causes voluntarily chosen (without manipulating a reference number of causes), and donation giving, along with GNP, while controlling for donors’ dispositional attitudes toward help giving (i.e., HAS).

### Materials and Methods

Participants were 95 students (*M*_*age*_ = 25.35, 61.7% female) recruited from a university online pool who voluntarily enrolled in this study in exchange for course credit and entered a raffle with three prizes of 50ILS each (equivalent to $15).

Participants were introduced to a description of the “CausePick” app, similar to studies 1 through 3. Participants were asked to assume they are entering the app for the first time and needed to indicate their charitable preferences from a list of 17 causes. Unlike studies 1 and 2 but similar to Study 3, participants were given the option to choose up to 7 causes they most care about. On the following page, participants indicated their intention to support a charitable campaign, assuming it fits their charitable preferences. Participants responded on a 7-point scale (1 = no chance I will donate, 7 = I will definitely donate). Next, participants indicated the amount they would be willing to donate to the campaign if they won prize money on a 50-point scale (0 = I will not donate any amount, 50 = I will donate all). On the page that followed, participants indicated their GNP with a single item as in Study 1, and completed the HAS scale (α = 0.77). Finally, participants indicated demographics such as age, gender, and mother tongue (For more details see Supplementary Appendix D).

### Results

Descriptive statistics and intercorrelations between the research variables are presented in Table 4. As seen in the table, the number of causes chosen by the participants was positively and significantly correlated with GNP (*r* = 0.34, *p* = 0.001) but not with willingness to donate (*r* = 0.18, *p* = 0.085) and donation amount (*r* = 0.05, *p* = 0.60). HAS was correlated with willingness to donate (*r* = 0.30, *p* = 0.003) but not with donation amount (*r* = 0.15, *p* = 0.13).

**TABLE 4:** Mean, std. deviation, and intercorrelations of study 4 variables (*N* = 95).

Variable	Mean	SD	1	2	3	4	5
Number of charitable causes chosen (up to 7)	5.88	1.52	−				
WTD (Single item; 1–7)	4.17	1.69	0.18	−			
Donation amount (0–$50)	24.33	15.33	0.05	0.61**	−		
GNP (Single item; 1–11)	7.40	2.54	0.34**	0.30**	0.37**	−	
HAS	3.95	0.38	0.09	0.30**	0.16	0.16	−

**p < 0.001.

**FIGURE 3:**
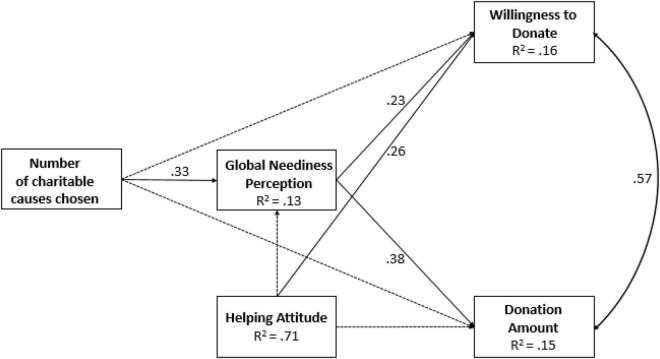
Path model from number of charitable causes chosen to willingness to donate and donation amount, mediated by GNP and controlled for HAS (Study 4). The values along the paths are standardized regression coefficients (betas), and correlation is shown along the double-arrow curve. The broken lines indicate statistically non-significant paths (*p* ≥ 0.05).

In a path model [χ^2^(0) = 0.00, see Figure 3], the indirect effect of the number of causes chosen, mediated by GNP, was in the expected direction yet did not reach significance for willingness to donate (*p* = 0.054) but did reach significance for the donation amount (*p* = 0.010).

Given that in this study causality cannot be inferred, we tested an alternative model in which the number of causes affected both willingness to donate and donation amount, with these two donation behavior variables in turn affecting GNP. In this path model, indirect effects on GNP of the number of causes chosen, mediated by the willingness to donate and donation amount, were not significant (willingness to donate: *p* = 0.857 and donation amount: *p* = 0.692). This provides additional evidence for the notion that GNP mediates the relationship between the number of causes chosen and donation giving.

### Discussion

The results of Study 4 replicate and add to the findings of Study 3 by showing that the relationship between the number of causes selected and willingness to support a campaign is driven by donors’ escalating sense of neediness in the world and not by their disposition toward helping. Although this disposition is related to help-giving to some extent, it is not influenced by the number of causes chosen and, hence, does not mediate the relationship.

In this study, the number of charitable causes did not relate directly to charitable behavior but only indirectly via GNP. This is somewhat inconsistent with the results found in our field data (Study 3), in which the number of charitable causes did relate to donors’ choice of a specific campaign—that is, to the willingness to donate (but not to the donation amount). One explanation could be that Study 4 employed a hypothetical general question to measure charitable behavior, which attenuated the potential relationship. In Study 3, donors viewed actual campaigns, while in Study 4 they were asked to consider donating to a campaign assuming it fit their charitable preferences but without viewing any information about an actual campaign. Another potential explanation is that Study 4 is underpowered to fully detect both the direct and indirect effects of the tested model. In the next study, we test these relations again with a larger sample.

In the final study, we sought to explore together the two forces that impact donation behavior. To that end, we manipulate the reference number of charitable causes donors can choose as important to them. Thus, some participants considered a small reference number (up to 3), while others considered a large reference number (up to 7), similar to studies 1 and 2. However, we did not confine participants to choosing an exact number, which allowed variations in the number of causes chosen as in studies 3 and 4. In line with our findings from previous studies, we expect that manipulation of the reference number will be negatively linked to charitable behavior; however, the actual number of causes participants voluntarily choose will be positively linked to charitable behavior, and the latter will be mediated by GNP. These effects will occur while controlling for participants’ attitudes toward helping.

## Study 5

In this study, we manipulate the reference number of causes donors can choose as important to them, but without forcing donors to choose a maximum number (i.e., participants can choose either up to 3 or up to 7 causes). In doing so, we allow for two opposing forces to shape donors’ behavior. The reference of a small or a large number of charitable causes is expected to drive behavior that follows the proportion dominance rationale. Thus, we hypothesize that participants with the smaller reference number (up to 3) will express more charitable behavior than participants with the larger reference number (up to 7). By enabling donors to freely choose up to the maximum number of causes presented, we allow for another process to take place. As the number of causes donors choose increases, the perception of global neediness escalates, prompting more charitable behavior. Thus, we hypothesize that the greater the number of causes participants choose (out of the maximum causes they can choose from), the greater their charitable behavior (i.e., willingness to donate and donation amount). We further hypothesize that this relationship will be mediated by GNP. We expect the hypothesized relationships to emerge while controlling for HAS.

### Materials and Methods

Five hundred participants (*M*_*age*_ = 36.67, 50.2% female) recruited from Prolific participated in this study in exchange for 0.5£ payment and the chance to win a $50 raffle prize.

As in our previous studies, participants read about the “CausePick” app. All participants were instructed to choose from a list of 17 causes the ones they most care about. Participants were randomly assigned to a different reference number of maximum causes from which they could choose. Participants were either instructed to choose up to 3 causes (small condition) or to choose up to 7 causes (large condition). This reference number served as our first independent measure. Unlike in studies 1 and 2, where participants were obligated to choose a fixed number, in this study they could “freely” choose causes (up to 3 or 7, depending on the condition). The actual number of charitable causes participants chose served as our second independent measure. On the following pages, participants indicated their willingness to donate, the amount they would be willing to donate if they won a $50 prize raffle, and their GNP (single item, as in studies 1 and 4). HAS was measured either at the beginning or the end of the survey as in studies 1 and 2 (α = 0.77). Finally, participants indicated demographics such as age, gender, and mother tongue (For more details, see Supplementary Appendix E).

### Results

We first conducted a *t*-test to compare the number of charitable causes chosen between the two reference number conditions. As expected, participants in the large condition chose more causes (*M* = 5.75, *SD* = 1.69) than those in the small condition [*M* = 2.96, *SD* = 0.24; *t*(498) = 25.55, *p* < 0.001], suggesting that the reference number manipulation was successful. We also tested whether the presentation order of HAS had an effect on the results, and, as in Study 1, it was non-significant, *p* = 0.48.

Descriptive statistics and intercorrelations between the research variables are presented in Table 5. As seen in the table, the reference number of charitable causes (the experimental manipulation) was negatively and significantly (*p* = 0.045) related to willingness to donate, and non-significantly related to the amount donated (*p* = 0.20) or to GNP (*p* = 0.13). The number of causes chosen was not related to the two dependent measures; willingness to donate (*p* = 0.33) and donation amount (*p* = 33) but was positively and significantly related to GNP (*r* = 0.13, *p* < 0.001).

**TABLE 5:** Mean, std. deviation, and intercorrelations of study 5 variables (*N* = 500).

Variable	Mean	SD	1	2	3	4	5	6
Reference number of charitable causes (0 = Three; 1 = Seven)	0.51	0.50	−					
Number of charitable causes chosen	4.37	1.85	0.75**	−				
WTD (Single item; 1–7)	4.46	1.67	−0.09*	0.04	−			
Donation amount (0–$50)	14.70	13.27	–0.06	0.04	0.36**	−		
GNP (Single item; 1–11)	3.83	1.09	–0.07	0.13**	0.26**	0.16**	−	
HAS	3.33	0.32	−0.10*	0.06	0.34**	0.29**	0.24**	−

*p < 0.05; **p < 0.001.

**FIGURE 4:**
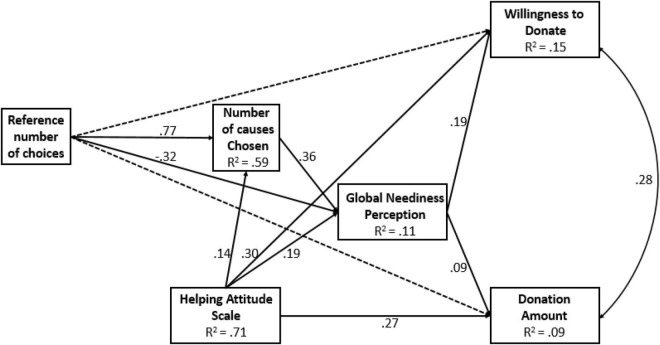
Path model from manipulation of reference number of charitable and number of charitable causes chosen to the willingness to donate and the donation amount, mediated by GNP, and controlled for HAS (Study 5). The values along the paths are standardized regression coefficients (betas), and correlation is shown along the double-arrow curve. The broken lines indicate statistically non-significant paths (*p* ≥ 0.05).

The hypothesized path model was tested and found to fit the data well, with χ^2^(2) = 2.71, *p* = 0.26 (see results in Figure 4).

The experimental manipulation of reference number of charitable causes was positively related to the actual number of causes chosen and negatively related to the GNP. The number of causes chosen was positively related to GNP, which, in turn, was positively related to the dependent variables: the willingness to donate and donation amount.

As part of the model, indirect (mediated) effects of experimental manipulation on the dependent variables were tested. In predicting willingness to donate, we found that reference number manipulation had a negative indirect effect mediated by GNP (β = −0.06, *p* = 0.001), and a positive effect mediated by number of causes chosen and then by GNP (β = 0.05, *p* = 0.001). The same pattern of mediation paths was found for the prediction of donation amount: a negative indirect effect of reference number manipulation mediated by GNP (β = −0.03, *p* = 0.049), and a positive effect mediated by number of causes chosen and then by GNP (β = 0.03, *p* = 0.046).

### Discussion

The results of Study 5 provide support for two opposing forces that shape charitable behavior. Results show that when the reference number of the maximum causes participants could choose was small, they expressed more charitable behavior than when the reference number was large. This result replicates the findings of studies 1 and 2 and is in line with proportion dominance reasoning, which suggests that contributing to one cause out of a small number of cases feels more meaningful than contribution to one cause out of a large number of causes. Results further show that the more charitable causes donors chose as important, the more global neediness they felt and the more charitable behavior they expressed, as indicated by greater willingness to donate and higher donation amounts. This result replicated the findings of studies 3 and 4.

## General Discussion

The prevalence of personalized charitable campaigns in the donation-raising arena has accustomed donors to pre-select their charitable preferences before making donation decisions.

Past research that has studied choice-driven effects in the donation setting has focused mainly on the choice of a donation recipient, either an individual person in need (e.g., Kogut and Ritov, 2005; Cryder et al., 2017; Ein-Gar et al., 2018; Bareket et al., 2022; Ein-Gar et al., 2022) or a charitable organization (e.g., Carroll et al., 2011; Soyer and Hogarth, 2011), thereby neglecting a pre-stage in which donors first choose the general charitable causes they care about. In this study, we focus on this unexplored stage of choosing charitable causes prior to deciding on a specific donation recipient. We demonstrate two opposing effects, showing how the process of choosing charitable causes donors care about influences their subsequent willingness to support a charitable campaign and their donation amount. We find that referencing donors to a larger rather than smaller number of causes reduces donation likelihood and donation amount (studies 1 and 2). We explain this effect based on the proportion dominance rationale, suggesting that helping one cause out of a small number of causes (Study 1: 4 causes; Study 2: 5 causes) feels more meaningful than helping one cause out of a large number of causes (Study 1: 8 causes; Study 2: 10 causes). Thus, the reference number of charitable causes has a negative effect on charitable behavior. We also find that as the actual number of charitable causes donors choose increases, so does their willingness to donate and to donate a larger amount (studies 3 and 4). This association is mediated by donors’ GNP (studies 4 and 5). Thus, the overall number of causes chosen has a positive relationship with charitable behavior. Finally, we find that the two effects can simultaneously influence charitable behavior (Study 5).^2^ In all studies testing path models (studies 1, 2, 4, and 5), HAS was a covariate; therefore, we test the direct and indirect paths between the model variables while accounting for individuals’ different attitudes toward help giving.

In this article, we introduce a new underlying mechanism that drives charitable behavior—namely, global need perception. We find that a small or large reference number of charitable causes does not influence GNP and that GNP does not mediate the effect of the reference number of charitable causes on charitable giving. However, we do find that GNP mediates the relationship between the number of charitable causes chosen and charitable giving. Furthermore, we find that GNP consistently correlated significantly positively with both willingness to donate and donation amount, regardless of whether GNP was measured with a single item (studies 1, 3, and 5) or 4 items (Study 5). In three studies (studies 1, 2, and 5), GNP correlated significantly positively with helping attitudes, while only in one study (Study 4) the relationship was not significant. In Study 2, we find that GNP correlated positively with fear of COVID-19, but to a lesser extent than its correlation with HAS. This pattern of results (and their magnitude) replicates previous findings we report in the Introduction. Finally, GNP was not found to relate to optimism. These results shed light on the convergent and discriminant validity of GNP.

### Limitations and Future Research

This article opens important new research avenues in the study of charitable behavior; however, it is not without limitations. From a methodological perspective, the studies were not preregistered, and some yielded relatively small effects. Future research should attempt to replicate the findings with more powered and pre-registered studies. Another limitation of this research was that, apart from Study 3, participants considered donating to a general hypothetical campaign. Future studies should test these predictions in situations when donors consider making an actual donation to a specific campaign. From a theoretical perspective, this research shows that different choice settings of charitable causes influence donations in opposing directions and may be driven by different underlying processes. However, we cannot infer causality between the number of charitable causes selected and donation behavior. Future research should provide causal evidence by varying the number of charitable causes selected and by manipulating the underlying driver of GNP. Furthermore, the choice-setting of the charitable causes was similar across studies. This means that in all studies participants saw a similar variety of causes, in a similar format. Future research should expand the scope of this investigation to other choice settings. For example, in this research, we did not explore the effect of choice-set size, or variety. Future research could test whether choosing a cause out of a choice set of 10 causes/charities or 50 causes/charities, all from similar or different domains changes charitable behavior. Furthermore, in our studies participants were asked to consider donating to a single campaign, which is a limitation, given that in reality they can donate to more than one campaign. Another interesting research direction would be to test whether the number of causes donors choose influences donors’ willingness to donate to several campaigns (as opposed to a single campaign).

Our research offers a new psychological driver for prosocial behavior—namely, GNP. However, we did not find that the reference number of charitable causes influences GNP. Future research can advance the exploration of this mechanism and its relation to the selection of charitable causes, identifying when and why GNP is heightened due to choosing charitable causes. Furthermore, given that this is a new individual difference, it is important that future research distinguish it from other individual differences and donation-related mechanisms. Our investigation was limited to attitudes toward helping, optimism, and fear of COVID-19. Future research could explore GNP’s relation to other individual differences such as perception of donation efficacy, self-signaling, and moral self. It also could identify other antecedents that impact the magnitude of GNP, such as mortality salience and types of charitable causes. Future research could also explore additional prosocial behaviors that may be driven by this mechanism—including volunteerism, advocacy of charitable campaigns, and even pro-environmental behaviors. Moreover, past research has shown that drivers enhancing a self-focused mindset reduce help giving (Levontin et al., 2015; Roux et al., 2015). This research has shown that drivers enhancing an other-giving mindset through GNP increase help giving. Future research can explore whether heightened GNP reduces self-focused behavior such as indulgent consumption and self-gifting.

In this research, we explore choice that does not directly influence the donation recipient (i.e., choice of charitable causes). Future research could explore how such initial choices have a downstream effect on choices that directly influence donation recipients. For example, past research has shown that choosing between two similar donation recipients leads to choice aversion (Ein-Gar et al., 2021). Future research can explore whether initially choosing a charitable cause reduces a donor’s tendency to opt-out of choosing a donation recipient. Intuitively, we assume that when individuals reflect on the charitable causes that are important to them, this reflection will in turn increase their willingness to help. Yet in this manuscript, we show that the effect of choosing charitable causes on donation giving is more complex than assumed. While the reference number of overall charitable causes may have a negative impact on donation giving, the actual number of charitable causes chosen has a positive impact on donation giving. These findings suggest that choosing charitable causes activates different motivational processes such as perceptions of proportion dominance and perceptions about the magnitude of neediness in the world.

### Practical Implications

This research is the first to show the important role that the pre-stage of selecting charitable causes has on donors’ subsequent behavior. One implication is that donation-raising agencies should consider the reference number they activate in donors’ minds when they ask them to choose a small or large number of causes. Our results suggest that smaller numbers would be more effective than larger numbers. Another implication is that asking donors to choose an exact number (e.g., choose 7 causes) or giving donors the option to choose causes with some variance (e.g, choose up to 7 causes) activates different mental processes and changes their donation decisions. Our results suggest that when donors are given choice variance, the more causes they choose, the greater their perception of global neediness in the world, and the more likely they are to donate. By designing the pre-registration stage in different ways, donation-raising agencies can decide whether they influence their prospect donors’ decisions through proportion dominance rationalizations or through neediness perception rationalizations.

## Data Availability Statement

The datasets presented in this study can be found in online repositories. The names of the repository/repositories and accession number(s) can be found below: https://osf.io/8gr6n/?view_only=b0703de9c9de4710ac3b48a85bbd54ec.

## Ethics Statement

The studies involving human participants were reviewed and approved by the Tel-Aviv University Ethics Committee. The participants provided their written informed consent to participate in this study.

## Author Contributions

DE-G designed studies 1, 2, 4, and 5 and collected the data. The studies were analyzed with the assistance of a statistical consultant (Dr. Ilan Roziner). The data of Study 3 was collected by AG and analyzed by DE-G. DE-G wrote the manuscript. AG read and approved the manuscript. Both authors contributed to the article and approved the submitted version.

## Conflict of Interest

The authors declare that the research was conducted in the absence of any commercial or financial relationships that could be construed as a potential conflict of interest.

## Publisher’s Note

All claims expressed in this article are solely those of the authors and do not necessarily represent those of their affiliated organizations, or those of the publisher, the editors and the reviewers. Any product that may be evaluated in this article, or claim that may be made by its manufacturer, is not guaranteed or endorsed by the publisher.

## Footnotes

1
https://web.archive.org/web/20180309140128/https:/www.causeisrael.org/

2 It should be noted that while one of the independent variables in our studies was experimentally manipulated (i.e., small vs. large reference number of charitable causes) and hence was causally related to the mediator (i.e., GNP) and dependent variables (i.e., willingness to donate and donation amount) in the tested models, the paths from the mediator to the dependent variables are only (partial) correlations, as suggested by Pieters (2017). Our measures may not fully comply with Pieters’ conditions for “meaningful” mediation analysis; nevertheless, we believe that our findings are at least not contradictory to interpretation in terms of mediation processes.
