# Supplementary material for: The Influence of Proportion Dominance and Global Need Perception on Donations
Source: Front Psychol. 2022 Jun 2;13:800867. doi: 10.3389/fpsyg.2022.800867 (PMC9202475; doi:10.3389/fpsyg.2022.800867)
Supplement: Supplementary file 1 [file Table_1.DOCX]

**Supplementary Material**

**Appendix A – Procedure Study 1**

**Participants**

Two hundred and seven students (*M_age_* = 24.7, 60.3% female) recruited from a university online pool participated in this study in exchange for course credit and entering a raffle with 20 prizes of 50ILS each (equivalent to $15).

**Procedure and Materials**

Half of participants first completed the HAS scale (page 1) while the other half completed the HAS scale at the end of the survey.

***Page 1: Platform Description***


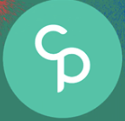
CausePick is an easy-to-use platform start-up for simple and easy donation giving.

Most people are interested in donating but don't want the hassle of searching for charitable causes, picking the best cause to support, and deciding each time how much to contribute. This platform solves all problems!

When you sign up for the first time, you specify the important causes you would like to support and a fixed monthly donation amount (say 20ILS per month) and of course provide payment details.

From that moment on everything is easy and convenient!


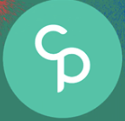
Each month, the platform sends 3 selected campaigns to your mobile, depending on which causes you marked as important

 
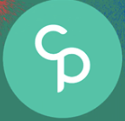
You select one campaign at the click of a button.

 
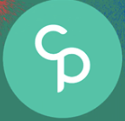
The platform makes sure the donation goes to the selected campaign.

 
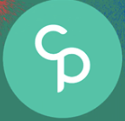
The platform sends you a summary invoice of all contributions at the end of the year.

easy. simple. and comfortable.

***Page 2: Reference Number of Charitable Causes manipulation***

Suppose you entered the
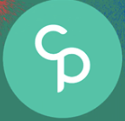
CausePick platform for the first time. Here are various donation topics. Please select **4** donation topics that you think are the most important (small condition).

Please select **8** donation topics that you think are the most important (large condition).

| 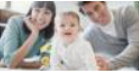  Babies and Parenting | 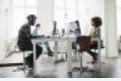  Start-ups |
| --- | --- |
| 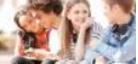  Youth | 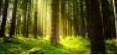  Environment |
| 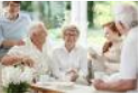  Elderly | 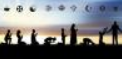  Religious |
| 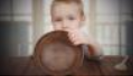  Hunger | 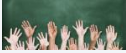  Human rights |
| 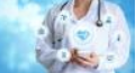  Health | 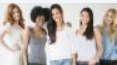  Women |
| 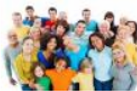  Community | 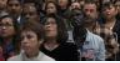Minorities and Immigrants |
| 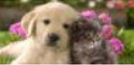  Animals | 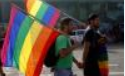  LGBT |
| 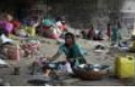  Poverty | 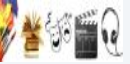  Culture and Arts |
| 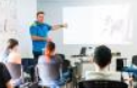  Education |  |

***Pages 3+4: Intention to Support a Campaign and Donation Amount***

The behavioral lab will be running a raffle to positively incentivize students to participate in experiments. The raffle prize will be 50ILS. If you draw the lottery and win the prize, would you be willing to donate the amount or part of it to a fundraising campaign related to one of the causes that you have identified as important?

| 1 | 2 | 3 | 4 | 5 | 6 | 7 |
| --- | --- | --- | --- | --- | --- | --- |
| No chance I will donate |  |  |  |  |  | I will definitely donate |

If you would consider making a donation, how much of the prize money would you give?

I will donate the entire amount

(50 ILS)

I will make no donation

(0 ILS)


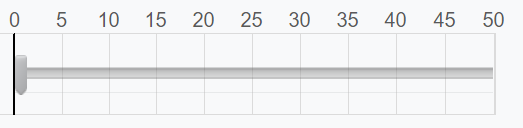


***Page 5: Global Neediness Perception***

Some people feel that there are many important social issues in the world that need the support of donations, while others feel that everything converges into a small number of important social issues that need charitable support. What, in your opinion, is the scope of all the social issues that need the support of donations in the world?

| 1 | 2 | 3 | 4 | 5 | 6 | 7 | 8 | 9 | 10 | 11 |
| --- | --- | --- | --- | --- | --- | --- | --- | --- | --- | --- |
| Extremely small/narrow |  |  |  |  |  |  |  |  |  | Extremely big/wide |

***Page 6: Helping Attitude Measure (half of participants completed it on page 1)***

This instrument is designed to measure your feelings, beliefs, and behaviors concerning your interactions with others. It is not a test, so there are no right or wrong answers. Please answer the questions as honestly as possible. Using the scale below, indicate your level of agreement or disagreement in the space which is next to each statement.

|  | Strongly Disagree | Disagree | Undecided | Agree | Strongly Agree |
| --- | --- | --- | --- | --- | --- |
| Helping others is usually a waste of time. |  |  |  |  |  |
| When given the opportunity, I enjoy aiding others who are in need. |  |  |  |  |  |
| If possible, I would return lost money to the rightful owner. |  |  |  |  |  |
| Helping friends and family is one of the great joys in life. |  |  |  |  |  |
| I would avoid aiding someone in a medical emergency if I could. |  |  |  |  |  |
| It feels wonderful to assist others in need. |  |  |  |  |  |
| Volunteering to help someone is very rewarding. |  |  |  |  |  |
| I dislike giving directions to strangers who are lost. |  |  |  |  |  |
| Doing volunteer work makes me feel happy. |  |  |  |  |  |
| I donate time or money to charities every month. |  |  |  |  |  |
| Unless they are part of my family, helping the elderly isn’t my responsibility. |  |  |  |  |  |
| Children should be taught about the importance of helping others. |  |  |  |  |  |
| I plan to donate my organs when I die with the hope that they will help someone else live. |  |  |  |  |  |
| I try to offer my help with any activities my community or school groups are carrying out. |  |  |  |  |  |
| I feel at peace with myself when I have helped others. |  |  |  |  |  |
| If the person in front of me in the check-out line at a store was a few cents short, I would pay the difference. |  |  |  |  |  |
| I feel proud when I know that my generosity has benefited a needy person. |  |  |  |  |  |
| Helping people does more harm than good because they come to rely on others and not themselves. |  |  |  |  |  |
| I rarely contribute money to a worthy cause. |  |  |  |  |  |
| Giving aid to the poor is the right thing to do. |  |  |  |  |  |

***Page 7: Demographics:***

Age, gender, mother tongue

***Results of analyses not reported in the main text:***

We tested the interaction effect of HAS presentation order and reference number of charitable causes condition on the two independent variables. We found no significant effects.

Willingness to donate: b = -.108; se = .073; t= -1.474; CI 95% [-.252, .036]

Donation amount: b = .343; se = .65; t= 0.528; CI 95% [-.936, 1.623]

We tested the path model from reference number of charitable causes to willingness to donate and donation amount, mediated by GNP and controlled for HAS *without* the two covariates of optimism and fear of Covid-19. The values along the paths are standardized regression coefficients (betas), and correlation is shown along the double-arrow curve. The broken lines indicate statistically non-significant paths (*p* >= .05). The path model (Mplus Version 8.6) of the theoretical relations between research variables fit the data well (Muthén & Muthén, 1998-2017), with χ2(0) = 0.00, p = .00.


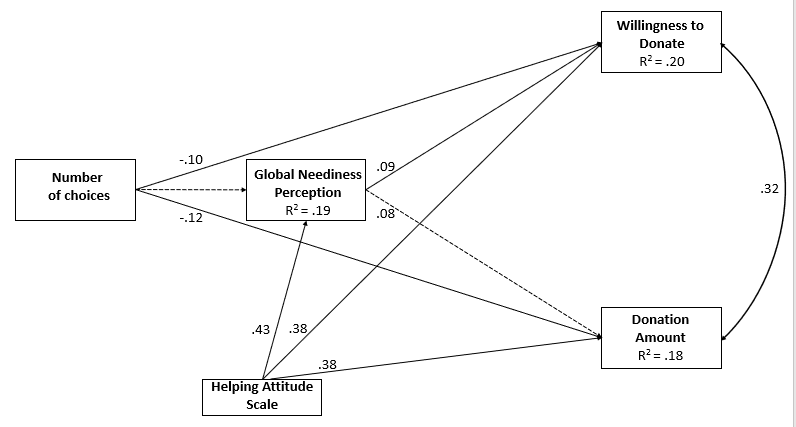


**Appendix B– Procedure Study 2**

**Participants**

Five hundred and one adults (*M_age_* = 38.8, 49.5% female) recruited through Prolific participated in this study in exchange for £0.8 payment and entering a raffle with a $20 prize.

**Procedure and Materials**

Half of participants first completed the HAS scale (page 1) while the other half completed the HAS scale at the end of the survey.

***Page 1: Platform Description – same as Study 1***

***Page 2: Reference Number of Charitable Causes manipulation***

Please select **5** donation topics that you think are the most important (small condition).

Please select **10** donation topics that you think are the most important (large condition).

***Pages 3+4: Intention to Support a Campaign and Donation Amount***

What are the chances that will you be willing to donate to one of the campaigns sent by the donation-raising App?

| 1 | 2 | 3 | 4 | 5 | 6 | 7 |
| --- | --- | --- | --- | --- | --- | --- |
| No chance I will donate |  |  |  |  |  | I will definitely donate |

For participation in the study, the behavioral lab will award a $20 prize out of all study participants. If you win the award, how much of the prize money would you consider donating to a campaign related to one of your chosen causes?

I will donate the entire amount

($20)

I will make no donation

($0)


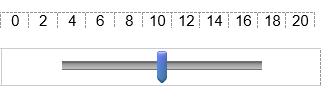


***Page 5: Global Neediness Perception (4 items***

Some people feel that there are many important social issues in the world that need the support of donations, while others feel that everything converges into a small number of important social issues that need charitable support. What, in your opinion, is the scope of all the social issues that need the support of donations in the world?

- Very few causes
- More than a few causes
- Several causes
- Quite a lot of causes
- Numerous causes

Please state how do you perceive the global neediness in the world today:

| 1 | 2 | 3 | 4 | 5 | 6 | 7 |
| --- | --- | --- | --- | --- | --- | --- |
| Almost no global neediness |  |  |  |  |  | Excessive global neediness |

In your opinion, what is the scope of social and environmental issues worldwide which require charitable support:

| 1 | 2 | 3 | 4 | 5 | 6 | 7 |
| --- | --- | --- | --- | --- | --- | --- |
| Few issues |  |  |  |  |  | Many issues |

What is your opinion about the social and environmental issues worldwide which require charitable support?

| 1 | 2 | 3 | 4 | 5 | 6 | 7 |
| --- | --- | --- | --- | --- | --- | --- |
| Insignificant issue/s |  |  |  |  |  | Significant issue/s |

***Page 6: Optimism scale***

Please indicate to what extent you agree or disagree with each of the following items.

|  | strongly disagree | disagree | neutral | agree | strongly agree |
| --- | --- | --- | --- | --- | --- |
| In uncertain times, I usually expect the best |  |  |  |  |  |
| It's easy for me to relax |  |  |  |  |  |
| If something can go wrong for me, it will |  |  |  |  |  |
| I'm always optimistic about my future |  |  |  |  |  |
| I enjoy my friends a lot |  |  |  |  |  |
| It's important for me to keep busy |  |  |  |  |  |
| I hardly ever expect things to go my way |  |  |  |  |  |
| I don't get upset too easily |  |  |  |  |  |
| I rarely count on good things happening to me |  |  |  |  |  |
| Overall, I expect more good things to happen to me than |  |  |  |  |  |

***Page 7: Fear of Covid-19 scale***

please indicate to what extent you agree or disagree with each of the following.

|  | strongly disagree | disagree | neutral | agree | strongly agree |
| --- | --- | --- | --- | --- | --- |
| I am most afraid of coronavirus-19 |  |  |  |  |  |
| It makes me uncomfortable to think about coronavirus-19 |  |  |  |  |  |
| My hands become clammy when I think about coronavirus-19 |  |  |  |  |  |
| I am afraid of losing my life because of coronavirus-19 |  |  |  |  |  |
| When watching news and stories about coronavirus-19 on social media, I become nervous or anxious |  |  |  |  |  |
| I cannot sleep because I’m worrying about getting coronavirus-19 |  |  |  |  |  |
| My heart races or palpitates when I think about getting coronavirus-19 |  |  |  |  |  |

***Page 8: Helping Attitude.*** Same as in Study 1 (half of participants completed it on page 1)

***Page9: Demographics:***

Age, gender, mother tongue

***Results of analyses not reported in the main text:***

We tested the interaction effect of HAS presentation order and reference number of charitable causes condition on the two independent variables. We found no significant effects.

Willingness to donate: b = -.003; se = .057; t= -0.05; CI 95% [-.114, .109]

Donation amount: b = .251; se = .221; t= 1.134; CI 95% [-.184, 0.685]

**Appendix C– Procedure Study 3**

**Participants**

Four hundred and eighty users entered the platform for the first time, 175 of them picked one out of three charitable campaigns, thereby indicating willingness to donate. Out of the 480 users 44 made an actual donation which varied between $0 to $108.

**Data collection**

CausePick (<https://web.archive.org/web/20180309140128/https:/www.causeisrael.org/>), a start-up online platform for raising donations, was established in November 2016 and was active until the end of 2018. Each month registered users received 3 charitable campaigns tailored to their pre-indicated charitable interests. Users picked one of the campaigns, and the fixed amount of donation they decided on at the initial registration stage was transferred to their chosen charity.

Our data includes 480 users who entered the platform between 7 July 2017 and 5 July 2018. Although the main goal of the platform was to encourage monthly donations to a different campaign each month, in this study we focused on users’ initial donation decision.

Users entering the platform for the first time read a description of the platform (step 1). Next, they selected the charitable causes they most care about from a list of 24 causes (step 2). Each cause was presented by a name and an icon, and users selected between 1 and 7 causes. Next, the platform's recommender system offered each user three personalized charitable campaigns from which users chose one (step 3). Next (step 4) users indicated the amount they wished to donate to their chosen campaign, which the platform set as the default amount for future donations (donors could change their default donation each month). In the final step, users provided the payment details. Below are illustrations of each step.

***Step 1: Description***


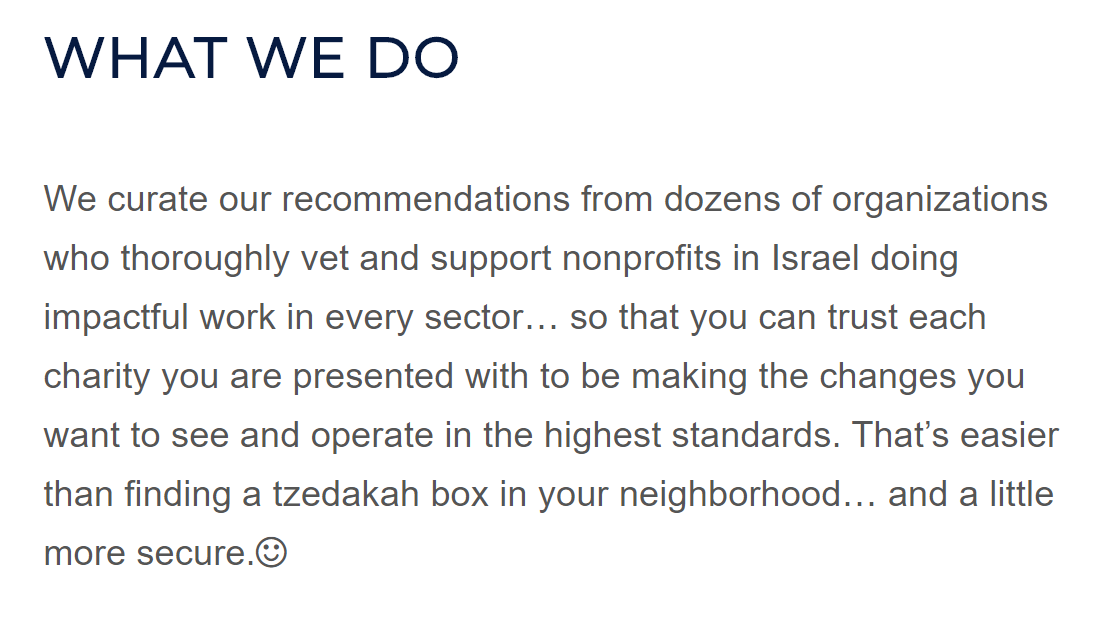


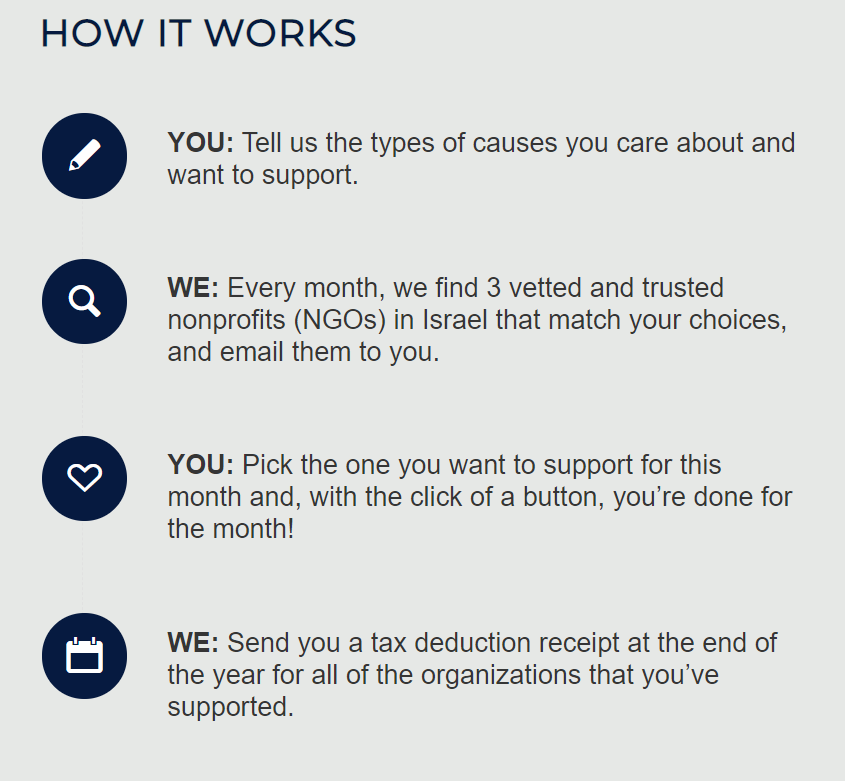


***Step 2: Cause selection*** (users can choose up to 7 topics)


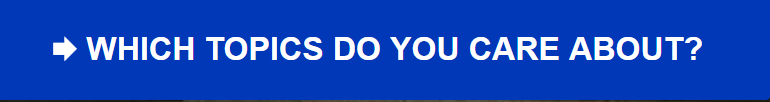


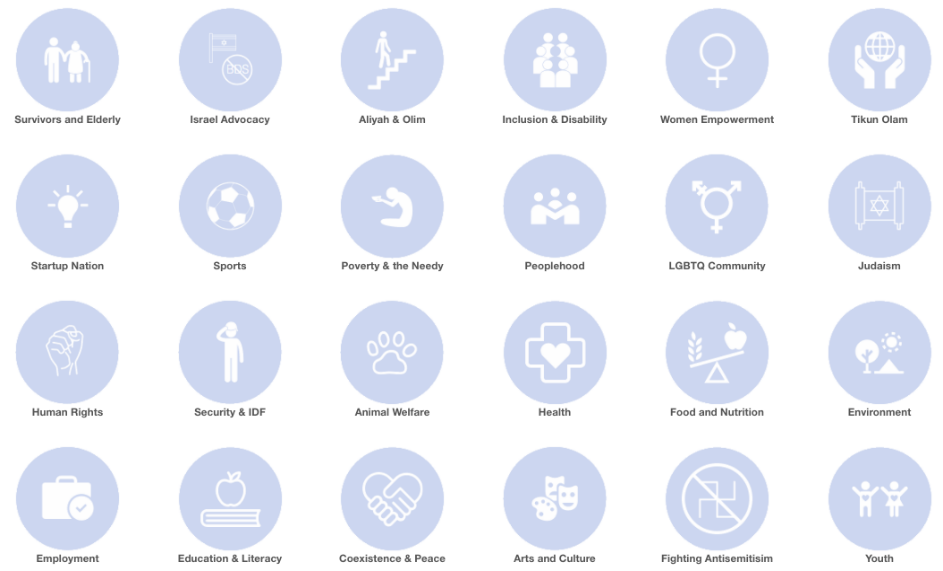


***Step 3: Campaign selection: An example***


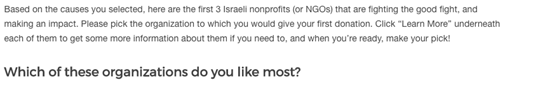


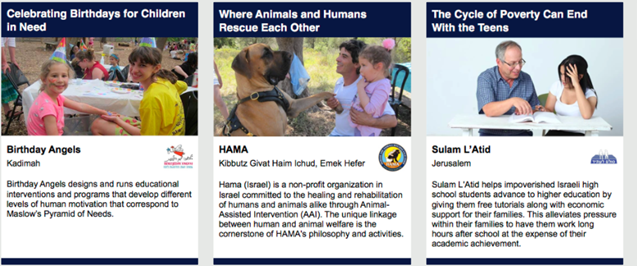


***Step 4: Donation amount choice***

**How strongly do you want to support Israeli charities like this one?**

Choose an amount to donate to the charity you just picked. We'll also set this as your donation level for organizations you pick in the future months.

(Don’t worry, you can change your level or cancel at any time.)

**Appendix D– Procedure Study 4**

**Participants**

Ninety-five students (M*_age_* = 25.35, 61.7% female) recruited from a university online pool participated in this experiment in exchange for entering a raffle with 3 prizes of 50ILS each (equivalent to $15).

**Procedure and Materials**

***Page 1: Platform Description*** - same as studies 1+2

***Page 2: Cause Selection*** (without a reference number manipulation)

Same as Studies 1+2 except that all participants were instructed to **select up to 7** topics

(Similar to study 3).

***Pages 3+4: Intention to Support a Campaign and Donation Amount*** - same as study 1


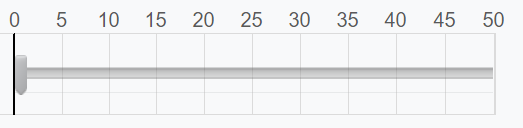


***Page 5: Global Neediness Perception*** – Same as study 1 (single item).

***Page 6: Helping Attitude Measure*** - Same as studies 1+2 (with randomized order)

***Page 7: Demographics:***

Age, gender, mother tongue

**Appendix E– Procedure Study 5**

**Participants**

Five hundred participants (*M_age_* = 36.67, 50.2% female) recruited from a Prolific participated in this study in exchange for 0.5£ payment and the chance to win a $50 raffle prize.

**Procedure and Materials**

***Page 1: Platform Description.*** Same as studies 1,2 & 4

Same as Study 2.

***Page 2: Cause Selection + reference number Manipulation***

**small condition**

Suppose you entered the 
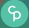
CausePick app for the first time. Here are various donation topics. Please select **up to 3** donation topics that you think are the most important.

**large condition**

Suppose you entered the 
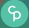
CausePick app for the first time. Here are various donation topics. Please select **up to 7** donation topics that you think are the most important.

***Pages 3-7: (Intention to Support a Charitable Campaign and Donation Amount, GNP***, ***Helping Attitude Measure***, ***Demographics)***

Same as Studies 1,2 &4.
